# Evolution of tigecycline- and colistin-resistant CRKP (carbapenem-resistant *Klebsiella pneumoniae*) in vivo and its persistence in the GI tract

**DOI:** 10.1038/s41426-018-0129-7

**Published:** 2018-07-09

**Authors:** Rong Zhang, Ning Dong, Yonglu Huang, Hongwei Zhou, Miaomiao Xie, Edward Wai-Chi Chan, Yanyan Hu, Jiachang Cai, Sheng Chen

**Affiliations:** 1 Second Affiliated Hospital of Zhejiang University, Hangzhou, China; 2 Shenzhen Key Lab for Food Biological Safety Control, Food Safety and Technology Research Center, Hong Kong PolyU Shenzhen Research Institute, Shenzhen, China; 3 State Key Lab of Chirosciences, Department of Applied Biology and Chemical Technology, The Hong Kong Polytechnic University, Hung Hom, Kowloon Hong Kong

## Abstract

Emergence of carbapenem-resistant *Klebsiella pneumoniae* (CRKP) strains that also exhibit resistance to tigecycline and colistin have become a major clinical concern, as these two agents are the last-resort antibiotics used for treatment of CRKP infections. A leukemia patient infected with CRKP was subjected to follow-up analysis of variation in phenotypic and genotypic characteristics of CRKP strains isolated from various specimens at different stages of treatment over a period of 3 years. Our data showed that (1) carbapenem treatment led to the emergence of CRKP in the gastrointestinal (GI) tract of the patient, which subsequently caused infections at other body sites as well as septicemia; (2) treatment with tigecycline led to the emergence of tigecycline-resistant CRKP, possibly through induction of the expression of a variant *tet(A)* gene located in a conjugative plasmid; (3) colistin treatment was effective in clearing CRKP from the bloodstream but led to the emergence of *mcr-1*-positive *Enterobacteriaceae* strains as well as colistin-resistant CRKP in the GI tract due to inactivation of the *mgrB* gene; and (4) tigecycline- and colistin-resistant CRKP could persist in the human GI tract for a prolonged period even without antibiotic selection pressure. In conclusion, clinical CRKP strains carrying a conjugative plasmid that harbors the *bla*_KPC-2_ and *tet(A)* variant genes readily evolve into tigecycline- and colistin-resistant CRKP upon treatment with these two antibiotics and persist in the human GI tract.

## Introduction

Multidrug-resistant bacterial pathogens that have emerged in the past decade are characterized by possession of extrachromosomal resistance elements that can undergo cross-species transmission, as well as expression of resistance mechanisms that render last-resort antibiotics, such as the carbapenems, ineffective for clinical applications^1^. Widespread dissemination of carbapenem-resistant *Enterobacteriaceae* (CRE) in clinical settings threatens to take medicine back to the preantibiotic era, and current efforts to invent and discover new antibiotics apparently have failed to reverse this ominous trend. To address this problem, there has been renewed interest in antibiotics such as the polymyxins, which have not been used clinically due to their relatively high toxicity^2,3^. However, this last hope has been tarnished by the recent discovery of a transmissible element that encodes resistance to polymyxins^4^. This element, a plasmid-borne *mcr-1* gene, was found to encode an enzyme that can modify the structure of lipid A in the molecule of lipopolysaccharide in Gram-negative bacteria, enabling them to exhibit resistance to polymyxins such as colistin. However, the potential risk of dissemination of the *mcr-1* gene among clinical strains is not clear. Apart from colistin, most clinical CRE strains exhibited high sensitivity to tigecycline. Although the exact molecular mechanism that renders tigecycline effective in treating infections caused by CRE is not well-defined, the drug has been widely used in combination with other antibiotics such as carbapenems to treat CRE infections in clinical settings, particularly in China, where colistin was not approved for clinical use and ceftazidime/avibactam was not available. In this study, we reported a comprehensive microbiological follow-up study of clinical strains recovered from a leukemia patient at various stages of treatment, including chemotherapy, hematopoietic stem cell transplantation, and recovery, over a period of 3 years. The data obtained provide insightful information on the evolution events that lead to the emergence of colistin- and tigecycline-resistant CRKP and the persistence of such strains in the gastrointestinal (GI) tract of the patient.

## Results

### Case description

A 29-year-old male patient was admitted to the second affiliated hospital of Zhejiang University, Hangzhou, China, in August 2014 with complaints of toothache and fever for over 1 week. A diagnosis of acute monocytic leukemia was made and the patient received chemotherapy and antimicrobial treatment with meropenem and vancomycin. Exhibiting signs of a lung infection on September 6, 2014, he was given a combination of meropenem and isepamicin for 5 days before being discharged from the hospital. The patient was readmitted for a second round of chemotherapy on September 29 and developed a fever, but was discharged upon treatment with meropenem, isepamicin, and vancomycin for 2 weeks. Blood and sputum cultures remained negative during this period (Table 1).

**Table 1:** Genetic and phenotypic characteristics of *K. pneumoniae* strains isolated from different specimen types at different dates

Strain ID	Source	Isolation date	Medical events	Antibiotics used	Prevailing symptoms	MIC (μg/ml)										MLST/PFGE
						IMP	MEM	FEP	FOS	AMK	LEV	CST	SCF	TIG	ISE	
KP1	Feces	20/11/14	Third chem	MEM, ISE	FEV	<0.5	<0.5	32	>256	<256	>256	0.5	<0.5	0.5	>128	ST1/KP1
KP2	Feces	28/11/14	Third chem	MEM, ISE, VAN	FEV, DIA	128	128	>256	>256	>256	256	0.5	>256	0.5	>128	ST11/KP2
KP3	Feces	05/05/15	TRAN	MEM, FOS, TIG	FEV, BDIA	128	128	>256	>256	>256	256	0.25	>256	0.5	>128	ST11/KP2
KP4	Blood	11/05/15	TRAN	MEM, FOS, TIG	HFEV, DIA	>128	>128	>256	>256	>256	256	0.25	>256	1	>128	ST11/KP2
KP5	Blood	18/05/15	TRAN	MEM, FOS, TIG	HFEV, DIA	128	64	>256	>256	>256	256	0.25	>256	1	>128	ST11/KP2
KP6	Blood	24/05/15	TRAN	MEM, FOS, TIG	HFEV, DIA	>128	128	>256	>256	>256	256	0.25	>256	1	>128	ST11/KP2
KP7	Feces	29/05/15	TRAN	MEM, IMP, TIG	FEV, LLA, DIA	64	32	>256	>256	>256	>256	0.5	256	1	>128	ST11/KP2
KP8	Blood, Feces	06/07/15	–	CST (IV)	FEV, DIA	64	64	128	>256	>256	>256	0.25	>256	1	>128	ST11/KP2
KP9	Sputum	27/10/15	–	MEM, LZD, CST	SU	128	128	>256	>256	>256	>256	0.25	256	**8**	>128	ST11/KP2
KP10	Feces	13/11/15	–	MEM, LZD, CST	SU	64	64	128	>256	>256	>256	**32**	256	**8**	>128	ST11/KP2
KP11	Sputum	04/11/15	Follow up	CST, PMB, MEM	PNEU, DIA	64	64	>256	>256	>256	>256	**32**	256	**4**	>128	ST11/KP2
KP12	Feces	10/10/16	Follow up	No	No	64	32	>256	>256	>256	>256	**32**	256	**4**	>128	ST11/KP2
KP13	Feces	16/02/17	Follow up	No	No	64	64	128	>256	>256	64	**32**	256	**4**	>128	ST11/KP2
KP14	Feces	05/05/17	Follow up	No	No	64	64	>256	>256	>256	64	**64**	256	**4**	>128	ST11/KP2
KP15	Feces	12/07/17	Follow up	No	No	64	32	>256	>256	>256	>256	**64**	256	**4**	>128	ST11/KP2
KP16	Feces	25/08/17	Follow up	No	No	64	64	>256	>256	>256	>256	**64**	256	**4**	>128	ST11/KP2
KP17	Feces	18/10/17	Follow up	No	No	64	64	>256	>256	>256	64	**64**	256	**8**	>128	ST11/KP2

Bolded values represent resistance to colistin or tigecycline. *chem* chemotherapy; *TRAN* hematopoietic stem cell transplantation, *FEV* fever, *HFEV* high fever, *BDIA* bloody diarrhea, *DIA* diarrhea, *PNEU* pneumonia, *LLA* liver lobe abscess, *SU* sacrococcygeal ulcer, *MEM* meropenem, *ISE* isepamicin, *VAN* vancomycin, *TIG* tigecycline, *FOS* fosfomycin, *IMP* imipenem, *CST* colistin, *LZD* linezolid, *PMB* polymyxin B, *FEP* cefepime, *AMK* amikacin, *LEV* levofloxacin, *SCF* cefperazone/sulbactam

**Fig. 1:**
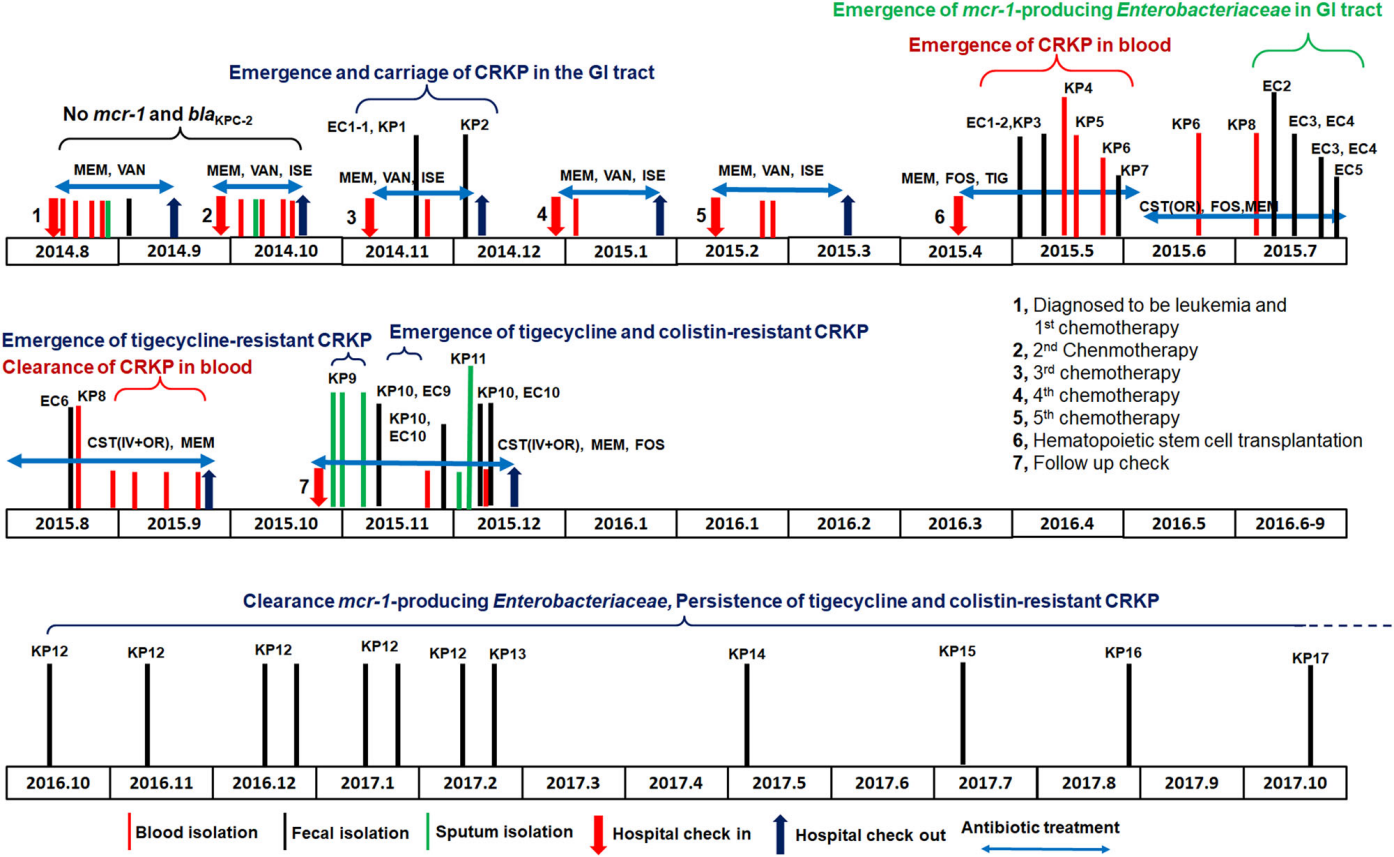
**Detailed outline of the disease process and emergence and clearance of** ***bla***_**KPC-2**_**-positive or** ***mcr-1*-positive** ***Enterobacteriaceae*** **strains in a leukemia patient.** Major medical events that occurred during a 2.5 years period are shown. Isolation of bacteria from different specimen types are labeled with different colored lines. The time points at which *bla*_KPC-2_-positive or *mcr-1*-positive *Enterobacteriaceae* strains emerged or cleared from different body sites are also stated, along with the duration and types of antibiotics used for treatment. All abbreviations are as explained in Tables 1 and 2

The patient received the third round of chemotherapy in November 2014. A week later, he developed a high fever (38.6 °C) and diarrhea and was again given a combination of meropenem and isepamicin. One *Escherichia coli* isolate, EC-1, and a *Klebsiella pneumoniae* strain, KP-Y1, both susceptible to carbapenems, were isolated from a diarrheal fecal sample (Table 1). The treatment regimen was then changed to meropenem, isepamicin, and vancomycin, and maintained for 4 days. Caspofungin was subsequently added and a carbapenem-resistant *K. pneumoniae* (CRKP) strain, KP2, was isolated for the first time from a fecal sample (Fig. 1, Table 1). The symptoms of fever and diarrhea persisted, and KP2-like strains remained detectable in fecal samples until early December of 2014. The patient was then given the fourth and fifth rounds of chemotherapy in December 2014 and February 2015, respectively. Symptoms were unremarkable except for intermittent fever and knee swelling. Prophylaxis with a combination of meropenem and isepamicin, followed by meropenem and vancomycin, continued for 3 weeks after each chemotherapy round. During this period, all culture findings remained normal.

The patient underwent hematopoietic stem cell transplantation in April 2015 and received prophylactic treatment with piperacillin-sulbactam. With fever and diarrhea developing soon afterwards, the treatment was reinforced by teicoplanin and posaconazole. A CRKP strain, KP3, was recoverable from a fecal sample. Diarrhea symptoms soon worsened, resulting in bloody stool and tenesmus. Meropenem treatment continued for 3 days before switching back to piperacillin-sulbactam and tigecycline. KP3 type strains remained detectable in stool, and a CRKP strain, designated as KP4, was isolated for the first time from blood in May 2015. The patient developed a high fever that reached 40 °C and was given high doses of tigecycline, isepamicin, and fosfomycin. Two more isolates, KP5 and KP6, were isolated from blood samples on the 18th and 24th of May 2015, respectively. A right liver lobe abscess was observed on May 29 when another fecal CRKP strain, designated KP7, was isolated (Fig. 1, Table 1). Antibiotics including meropenem, tigecycline, amikacin, and voriconazole were provided but were ineffective in relieving symptoms. The patient then developed fever, diarrhea and pan-resistant sepsis. The hospital ran out of antibiotic choices and the patient was subsequently treated with tigecycline and caspofungin, which were supplemented with colistin purchased from overseas. Tigecycline was given to the patient with 100 mg delivered IV every 12 h from May 8, 2015, through June 3, 2015.

The combined use of colistin and multiple antibiotics intravenously and orally eventually eased the diarrheal symptom, but could not immediately clear CRKP from the bloodstream. Perianal pain, fever, and signs of liver abscess persisted in the following weeks. Colistin treatment continued until September 1, 2015, and then treatment was switched to polymyxin B. CRKP remained detectable in fecal samples and occasionally in blood until early September, when blood samples became sterile, indicating that colistin was eventually effective in eradicating CRKP strains (Fig. 1). On the other hand, *mcr-1*-positive, colistin-resistant strains of *E. coli*, *Morganella morganii*, and *Citrobacter freundii* were consecutively isolated from fecal samples from July 13, 2015, onwards (strains EC2-7), indicating that colistin selected its own resistance during the treatment process (Fig. 1, Table 1).

Seven months after the transplant, an ulcer was observed at the sacrococcygeal site. Meropenem and linezolid treatment was initiated, followed by colistin, fosfomycin (IV), and then polymyxin B (IV). Colistin treatment was started with polymyxin B with 1 million units IV every 8 h and then changed to colistin with the same dose from June 3, 2015 to November 30, 2015. During treatment, CRKP was detectable in sputum, and both CRKP and *mcr-1-*positive *E. coli* were recoverable from fecal samples in retrospective analysis. However, blood samples remained sterile. In early December of 2015, pneumonia and GI tract symptoms appeared, prompting intravenous and oral administration of polymyxin B and E. CRKP and *mcr-1*-positive *E. coli* strains continued to be detectable in fecal samples and CRKP strains could be recovered occasionally in sputum but not blood. The symptoms began to resolve a few days later and the patient was discharged on December 15, 2015, with carriage of CRKP and *mcr-1*-positive, colistin-resistant *E. coli* in stool. CRKP isolated from sputum and a fecal sample during this period were selected for further study (strains KP9 and KP10, respectively). Two *mcr-1* positive *E. coli* isolates, which were recovered from fecal samples collected in November 2015 and designated EC-Y9 and EC-Y10, respectively, were also subjected to microbiological and genetic characterization (Fig 1, Table 1).

The patient recovered gradually and remained healthy since being discharged from the hospital. To determine if *mcr-1*-positive *Enterobacteriaceae* and CRKP persisted in his GI tract, we performed fecal isolation for these organisms in October 2016. CRKP strains, but not *mcr-1*-positive *Enterobacteriaceae*, were recoverable from fecal samples. We designated these CRKP strains as KP12–KP17, respectively. Another seven follow-up procedures were performed, and similar results were obtained (Fig. 1, Table 1).

### Acquisition of *bla*_KPC-2_-bearing *K. pneumoniae* and evolution of tigecycline and colistin resistance in vivo

*K. pneumoniae* strains collected during the study period were subjected to characterization of their antimicrobial susceptibility to various antibiotics, sequence types (STs), PFGE pattern, and carriage of antibiotic resistance genes. The first *K. pneumoniae* strain isolated from the patient, KP1, was found to be susceptible to carbapenems and belong to ST1. However, strains KP2 through KP17 were all resistant to carbapenems and found to belong to ST11, the most common ST of CRKP in the hospital environment, indicating that such strains were likely to be hospital-acquired upon treatment with carbapenems. All CRKP strains tested exhibit highly similar PFGE profiles and harbored the *bla*_KPC-2_ and *bla*_CTX-M-9_ elements, confirming that a single strain was responsible for the infection. S1-PFGE and Southern hybridization confirmed that the *bla*_KPC-2_ gene was located in a ~ 60-kb plasmid. Our findings show that once emerged in the GI tract, the CRKP strains were already resistant to multiple antibiotics and not readily eradicated even with intensive and prolonged antimicrobial treatment, which resulted in further enrichment of CRKP strains within the gut microbiome and enhancement of the risk of untreatable opportunistic infections in the immunocompromised patient.

Importantly, strain KP2, which was first isolated from a fecal sample of the patient, ended up causing septicemia (KP4) 6 months later when the patient was subjected to hematopoietic stem cell transplantation, suggesting that CRKP in the GI tract would readily cause infections in other sites of the body when the host defense system is weakened (Table 1). Upon treatment with tigecycline for several months before using colistin, CRKP strains remained in the bloodstream. Our data showed that the use of tigecycline actually resulted in the selection of a tigecycline-resistant CRKP strain (KP9), which has persisted in the GI tract of the patient ever since (Table 1). According to the clinical record, CRKP strains in the bloodstream could only be cleared by a high dose of colistin IV, without which the patient would have died of septicemia. This scenario is commonly seen among intensive care unit patients and entails development of novel intervention strategies^5–7^. The use of colistin treatment indeed cleared CRKP from the bloodstream, however, such a strain remained detectable in fecal samples from the patient. Interestingly, upon the use of colistin, the tigecycline-resistant CRKP strain further evolved to become resistant to colistin, with KP10 being the first such strain recovered from the patient. This tigecycline- and colistin-resistant CRKP strain has persisted in the GI tract of the patient for more than 2 years (Fig. 1).

### Characterization of *mcr-1*-bearing *Enterobacteriaceae*

Another interesting observation is that 4 weeks after continuous treatment with colistin, *mcr-1*-positive *Enterobacteriaceae* strains became detectable in fecal samples, presumably due to selection of a preexisting resistant subpopulation or acquisition of these strains from other resources (Table 2). Since then, *mcr-1*-positive *Enterobacteriaceae* strains remained recoverable from fecal samples, inferring that long-term usage of colistin can indeed enrich *mcr-1*-positive *Enterobacteriaceae* strains in the GI tract. This idea is consistent with a previous finding that *mcr-1*-positive *Enterobacteriaceae* are detectable in human gut microflora, including that of children^8^. The nine *mcr-1* positive *Enterobacteriaceae* strains tested included seven *E. coli* strains and one each of *M. morganii* and *C. freundii*. Importantly, these *mcr-1*-bearing strains were susceptible to most of the antibiotics tested except fluoroquinolones, suggesting that *mcr-1* had not been acquired by CRKP or other CRE strains during colistin treatment. The seven *mcr-1*-positive *E. coli* strains belonged to four ST, with ST58 being the most dominant. However, different PFGE patterns were detectable, with three of the seven *mcr-1*-positive *E. coli* strains being untypable, indicating that the *mcr-1* element had been disseminated to multiple bacterial hosts in the patient’s body (Table 2). Conjugation experiments showed that eight out of the nine strains could transfer their colistin resistance phenotype to the *E. coli* J53 recipient strain. This finding confirms that the colistin-resistance determinant was located in a transmissible plasmid. S1-PFGE and Southern hybridization using a *mcr-1* probe showed that such plasmids belonged to two major types of ~30 kb and ~60 kb in size, suggesting that the gene can be incorporated into various extrachromosomal elements that are transmissible among *Enterobacteriaceae* strains in the human GI tract. The ~30-kb and ~60-kb *mcr-1*-positive plasmids were selected for sequencing analysis, with results showing that the ~33-kb conjugative plasmid was highly similar to one harbored by an *E. coli* strain recovered from farm animals in Estonia (NCBI accession no. KU743383), as well as other plasmids derived from *E. coli* of animal origin (pECJC-B65-33), whereas the ~60-kb, *mcr-1*-bearing plasmid was similar to the original *mcr-1*-bearing plasmid pHNSHP45^4^. The data suggested that these two *mcr-1*-carrying plasmids were readily transmitted among *Enterobacteriaceae*. Nine months after the discharge of the patient (from December 2015 until September 2016), the *mcr-1*-positive *Enterobacteriaceae* strains could not be recovered from the GI tract of the patient, suggesting that without colistin selective pressure, *mcr-1*-positive *Enterobacteriaceae* could be reduced to an undetectable level.

**Table 2:** Genetic and phenotypic characteristics of *E. coli* isolated from fecal samples at different dates and the corresponding transconjugants that have acquired the *mcr-1*-borne plasmid (strain number with a suffix of C)

Strain ID	Isolation date	Species	AMR genes	*mcr-1* plasmids	MIC (μg/ml)									MLST	PFGE
					IMP	MEM	FEP	CAZ	AMK	LEV	CST	SCF	ATM		
EC-J53	–	*E. coli*	–	–	≤0.25	≤0.25	≤0.5	≤0.5	1	≤0.125	1	≤0.5	≤0.5		
EC1–1	20/11/2014	*E. coli*	–	–	<0.25	<0.25	<0.5	<0.5	4	128	0.25	2	<0.5	224	EC1
EC1–2	09/05/2015	*E. coli*	–	–	<0.25	<0.25	<0.5	<0.5	4	128	0.25	1	<0.5	224	UT
EC2	13/07/2015	*E. coli*	*mcr-1*	N/A	0.5	0.25	<0.5	<0.5	2	8	8	<0.5	<0.5	58	EC2
EC2C		EC-J53	*mcr-1*	~33 kb	≤0.25	≤0.25	≤0.5	≤0.5	1	≤0.125	8	≤0.5	≤0.5		
EC3	15/07/2015	*E. coli*	*mcr-1*	N/A	<0.25	<0.25	<0.5	<0.5	>256	4	8	<0.5	<0.5	607	EC3
EC3C		EC-J53	*mcr-1*	~33 kb	≤0.25	≤0.25	≤0.5	≤0.5	1	≤0.125	8	≤0.5	≤0.5		
EC4	20/07/2015	*M. morganii*	*mcr-1*		<0.25	<0.25	<0.5	<0.5	1	4	>64	2	<0.5	58	NT
EC5	24/07/2015	*C. freundii*	*mcr-1*		0.5	0.25	<0.5	<0.5	4	64	16	1	<0.5		NT
EC5C		EC-J53	*mcr-1*	~ 60kb	≤0.25	≤0.25	≤0.5	≤0.5	1	≤0.125	4	≤0.5	≤0.5		
EC6	10/08/2015	*E. coli*	*mcr-1*		<0.25	<0.25	<0.5	<0.5	2	8	8	<0.5	<0.5	58	EC4
EC6C		EC-J53	*mcr-1*	~ 33kb	≤0.25	≤0.25	≤0.5	≤0.5	1	≤0.125	8	≤0.5	≤0.5		
EC7	18/08/2015	*E. coli*	*mcr-1*		<0.25	<0.25	<0.5	<0.5	8	8	8	<0.5	<0.5	58	UT
EC7C		EC-J53	*mcr-1*	~ 33kb	≤0.25	≤0.25	≤0.5	≤0.5	1	≤0.125	8	≤0.5	≤0.5		
EC8	18/08/2015	*E. coli*	*mcr-1*		<0.25	<0.25	<0.5	<0.5	8	2	8	<0.5	<0.5	46	EC5
EC8C		EC-J53	*mcr-1*	~ 33kb	≤0.25	≤0.25	≤0.5	≤0.5	1	≤0.125	8	≤0.5	≤0.5		
EC9	13/11/2015	*E. coli*	*mcr-1*		<0.25	<0.25	>256	>256	>256	128	8	4	32	3944	UT
EC9C		EC-J53	*mcr-1*	~ 60kb	≤0.25	≤0.25	≤0.5	≤0.5	1	≤0.125	8	≤0.5	≤0.5		
EC10	25/11/2015	*E. coli*	*mcr-1*		<0.25	<0.25	>256	>256	>256	128	8	4	32	3944	UT
EC10C		EC-J53	*mcr-1*	~ 33kb	≤0.25	≤0.25	≤0.5	≤0.5	1	≤0.125	8	≤0.5	≤0.5		

*AMR* antimicrobial resistance genes, *IPM* imipenem, *MEM* meropenem, *FEP* cefepime, *CAZ* ceftazidime, *AMK* amikacin, *LEV* levofloxacin, *CST* colistin, *SCF* cefperazone/sulbactam, *ATM* aztreonam, *UT* untypable, *NT* not determined

### Mechanism of tigecycline and colistin resistance in CRKP

**Fig. 2:**
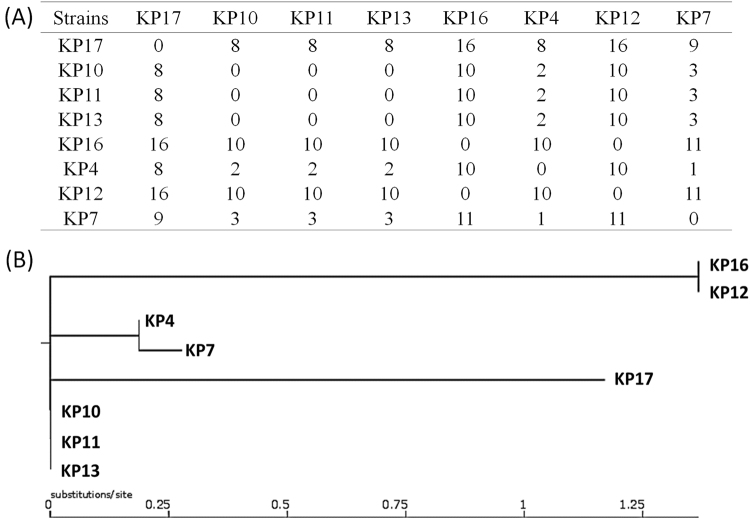
**Phylogenetic analysis of carbapenem-resistant** ***K. pneumoniae*** **strains isolated from the patient at different stages of infection.** (**a**) Pairwise SNP analysis of the test strains. (**b**) Phylogenetic tree depicting the genetic relatedness of the strains

**Fig. 3:**
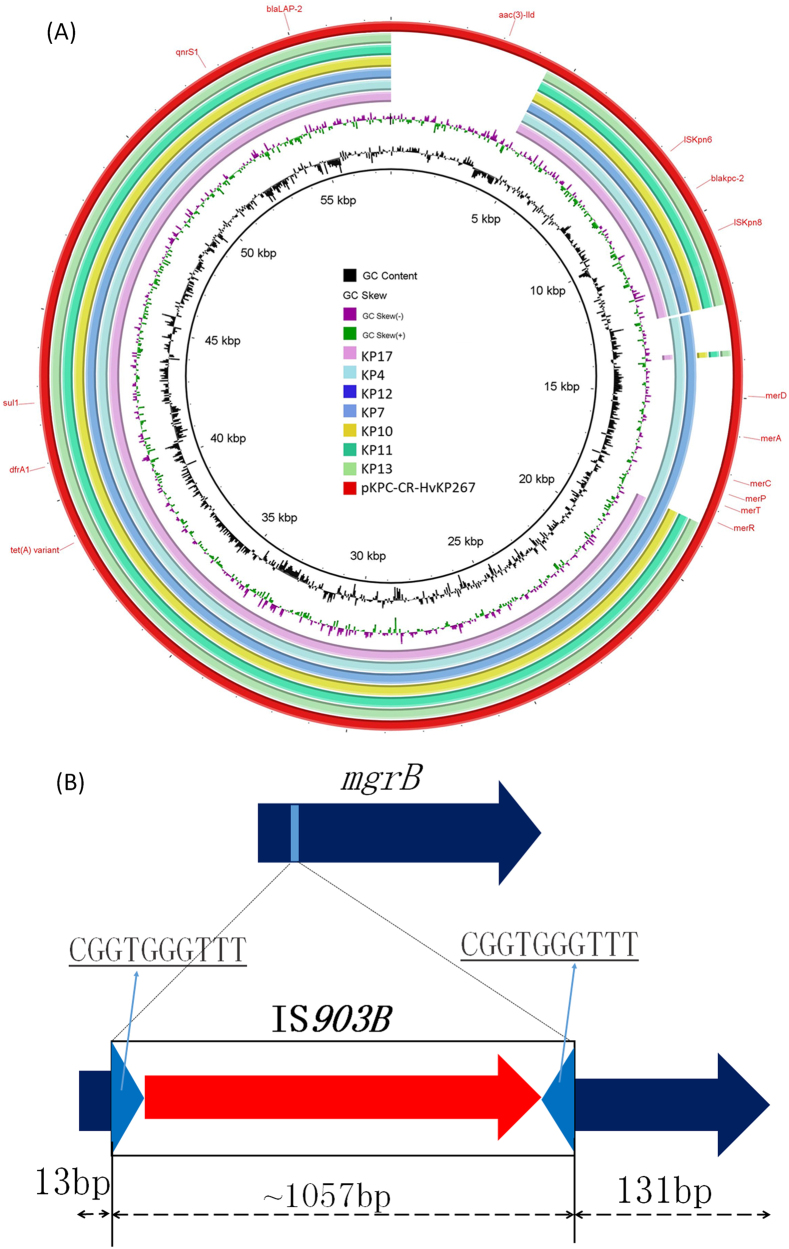
**Mechanisms of tigecycline and colistin resistance in clinical CRKP**. (**a**) pKPC-CR-HvKP267-like plasmid carrying both *tet(A)* and *bla*_KPC-2_ genes was detectable in CRKP strains isolated in this study. (**b**) Insertion of IS*903B* into the 13th and 14th position of the *mgrB* gene

To further investigate mechanisms underlying the development of tigecycline and colistin resistance in CRKP, whole-genome sequencing (WGS) was performed on eight strains collected at different stages, namely KP4 (Accession no.: SAMN08725539), KP7 (Accession no.: SAMN08725538), KP10 (Accession no.: SAMN08725534), KP11 (Accession no.: SAMN08725535), KP12 (Accession no.: SAMN08725536), KP13 (Accession no.: SAMN08725537), KP16 (Accession no.: SAMN08725533), and KP17 (Accession no.: SAMN08725532) (Table 1, Fig. 2). Phylogenetic analysis of these strains showed that they indeed belonged to the same genetic clone, with the maximum genetic difference being less than 1.5% (Fig. 2a). Pairwise SNP analysis between these strains showed that the maximum number of SNPs was 16, confirming that they belong to the same clone. It was recently reported that tigecycline resistance in *K. pneumoniae* was due to carriage of a *tet(A)* variant in a conjugative plasmid, pKPC-CR-HvKP267 (MG053313), which also contained a *bla*_KPC-2_-bearing element^9^. Alignment of the Illumina reads of these strains against this plasmid demonstrated that these strains all carried a similar type of plasmid that lacked a fragment containing the an *aac(3)-IId* gene when compared to pKPC-CR-HvKP267. Several plasmids also lacked the *mer* gene cluster. However, all plasmids were found to carry the *bla*_KCP-2_ gene and a *tet(A)* variant identical to that harbored by pKPC-CR-HvKP267. Interestingly, strains KP4 and KP7 were susceptible to tigecycline, yet harbored the same plasmid and the *tet(A)* variant, suggesting that the *tet(A)* gene was not expressed under normal conditions but inducible upon prolonged treatment with tigecycline (Fig. 3a).

CRKP strain KP10 and subsequently recovered strains became colistin resistant due to the prolonged use of colistin in the patient. It has been reported that colistin resistance in *K. pneumoniae* can be mediated by inactivation of the *mgrB* gene through insertion of insertion sequence (IS)^10^. Genome analysis of these strains showed that strains KP10 through KP17 carried an IS*903B* element between the 13th and 14th base pair of *mgrB*, yet the *mgrB* gene in strains KP4 and KP7, which were susceptible to colistin, was not inactivated by an IS element. These findings show that prolonged use of colistin could induce colistin resistance through inactivation of the *mgrB* gene (Fig. 3b). All CRKP strains were analyzed for the presence of virulence plasmids, as reported in our previous study^11^, and our results showed that none of the CRKP strains carried virulence plasmids (data not shown).

## Discussion

This report, which describes a long battle against a series of bacterial infections that occurred in an immunocompromised patient, provides insightful information regarding the choice of antibiotics in the management of infections caused by CRKP and the possible treatment outcomes. Our data indicate that colonization and enrichment of CRKP in the human GI tract have become common events, presumably due to frequent episodes of antibiotic treatment, which results in a significantly higher risk of untreatable opportunistic and systemic infections in cases in which the host defense system has weakened. To date, carriage of CRKP in the GI tract of hospitalized patients has become commonplace^5,12,13^. Such strains are readily acquired in the hospital environment and further selected upon antibiotic treatment, thereby contributing to further transmission of CRKP in clinical settings. The GI tract of patients could be considered as a reservoir and incubator of CRKP, and should therefore be regarded as a target of intervention in the development of novel hospital infection control policies. Most importantly, the CRKP strains located in the GI tract could be transmitted to other organs such as the lungs, and cause infection or even septicemia under various circumstances such as during surgery or chemotherapy.

Choices for treatment of CRKP infections are extremely limited. A high dose of carbapenems is still considered as one therapeutic option despite minimal effectiveness. As most clinical CRKP strains exhibit sensitivity to tigecycline in in vitro minimum inhibitory concentration (MIC) tests, this drug is widely used in combination with carbapenem in the treatment of CRKP infections. However, our data showed that combined treatment of CRKP bloodstream infection with tigecycline and other antibiotics such as carbapenems was not particularly effective in clearing CRKP from the bloodstream, which was consistent with several earlier reports^14,15^. Furthermore, treatment with tigecycline could lead to induction of *tet(A)* gene expression in tigecycline-susceptible CRKP strains that carry the *tet(A)* variant, leading to the development of tigecycline resistance. The *tet(A)* gene was found in a conjugative plasmid that also harbored the *bla*_KPC-2_ gene. This plasmid is commonly harbored by clinical CRKP strains; hence, development of tigecycline resistance readily occurs among such strains upon tigecycline treatment. On the other hand, our data showed that intravenous treatment with colistin could effectively eradicate CRKP from blood. However, the use of colistin could also lead to the development of colistin-resistant CRKP strains through IS element-mediated inactivation of the *mgrB* gene. These tigecycline- and colistin-resistant CRKP strains cannot be cleared by colistin in the human GI tract and can persist for a long period even without antibiotic selection pressure. The role of colistin in treating CRKP infections was limited as resistance to this antibiotic readily develops and persists. In China, the colistin resistance rate among clinical CRKP strains remains relatively low, presumably because colistin has not been approved for clinical use in China until recently. The rate of colistin resistance in clinical CRKP is worthy of close monitoring as colistin was approved for clinical use in early 2018. Although prolonged usage of colistin could lead to selection of *mcr-1-*positive, colistin-resistant *Enterobacteriaceae* strains, such strains appear to be confined to the GI tract and rarely cause infections in other organs; this idea is consistent with findings in the current study in that septicemia caused by *mcr-1*-bearing *Enterobacteriaceae* strains is rare^16^. Nevertheless, since *mcr-1*-bearing CRKP strains have been reported by other studies^17–20^, the clinical value of colistin as a last-resort antimicrobial agent is expected to erode rapidly. Another choice of treatment for CRKP is ceftazidime-avibactam, which has been shown to be effective in treating CRKP infection^21,22^. However, this antibiotic is currently unavailable in China and its efficacy in clearing CRKP in this patient was not tested.

Phylogenetic analysis showed that the majority of CRKP strains tested in this study were genetically related. A significant proportion of such strains were also found to harbor structurally similar conjugative plasmids that contained the *tet(A)* and *bla*_KPC-2_ genes, suggesting that tigecycline or colistin treatment of infections caused by these strains would significantly enhance development of tigecycline and/or colistin resistance among these strains. A comprehensive surveillance program also needs to be established to monitor the variation of the prevalence of these CRKP strains and their impact on human health. New therapeutics to combat infections caused by these types of strains are also urgently required.

## Materials and methods

### Bacterial strains

Bacteria in blood and sputum samples were isolated according to standard procedures. The isolation of *mcr-1*-bearing *Enterobacteriaceae* strains from fecal samples was performed as described previously^23^. The isolation of carbapenem-resistant *Enterobacteriaceae* from fecal samples was performed as follows. Approximately, 5 g of feces from the patient was spread onto *Salmonella*-*Shigella* agar plates supplemented with 0.5 μg/ml meropenem for 18 h; more than 20 single colonies were picked for further purification and detection of carbapenemase genes by polymerase chain reaction (PCR) as previously described^24^. All *mcr-1*-positive and carbapenem-resistant *Enterobacteriaceae* isolates were identified using the Vitek 2 system (bioMérieux, Marcy-l’E´ toile, France) and confirmed by a MALDI-TOF MS apparatus (Bruker Microflex LT, Bruker Daltonik GmbH, Bremen, Germany).

### Antimicrobial susceptibility testing

The MICs for indicated antibiotics, as shown in Tables 1 and 2, were determined using the agar dilution method. The results were analyzed according to the CLSI criteria of 2016^25^. MICs for colistin and tigecycline were determined using the broth dilution method. The 2014 EUCAST breakpoints were used (available at http://www.eucast.org/clinical_breakpoints/) for tigecycline.

### Screening of β-lactamase genes and the *mcr-1* gene

PCR and nucleotide sequencing were employed to screen for the carbapenemase-encoding genes *bla*_VIM_, *bla*_IMP_, *bla*_KPC_, *bla*_OXA-48_, and *bla*_NDM-1_ as well as the extended-spectrum β-lactamase genes *bla*_CTX-M_, *bla*_TEM_ and *bla*_SHV_, as described previously^26^. Screening of *mcr-1* was performed as previously described^23^.

### PFGE, S1-PFGE, conjugation, Southern hybridization, and MLST typing

Pulsed-field gel electrophoresis (CHEF-MAP-PER System, Bio-Rad Laboratories, Hercules, CA, USA) was used to assess the genetic relatedness between the test isolates and the corresponding transconjugants as described previously^27^. Conjugation experiments, S1-PFGE and Southern hybridization were performed as previously described^24^. MLST targeting seven housekeeping genes was performed in *bla*_NDM-1_-bearing *E. coli*, *K. pneumoniae*, and *E. cloacae* isolates, using primers listed in online databases (http://pubmlst.org/ecloacae/ for *E. cloacae*, http://bigsdb.web.pasteur.fr/klebsiella/klebsiella.html for *K. pneumoniae* and http://mlst.warwick.ac.uk/mlst/dbs/Ecoli for *E. coli*). The resultant PCR products were purified and sequenced. STs were assigned using online database tools.

### WGS and comparative genomics analysis

Genomic sequencing was performed by means of the Illumina NextSeq 500 sequencing platform. Raw Illumina reads were trimmed or filtered to remove low-quality sequences and adapters, and assembled *de novo* by the SPAdes Genome Assembler v3.9.1^28^. The draft genomes were annotated with the RAST tool^29^ and Prokka^30^. Genome sequence comparison was performed using the BLAST Ring Image Generator (BRIG)^31^. Pangenome analysis was conducted using the Roary Pangenome analysis pipeline based on the Prokka annotation^32^. SNPs were determined by the CSI Phylogeny 1.4 [1] pipeline available from the Center for Genomic Epidemiology (www.genomicepidemiology.org), using default settings and WGS raw reads. Capsular typing was performed on the assembled sequences using Kaptive^33^. The genome sequences were deposited into GenBank.
